# Immunotherapy and Immune Infiltration in Patients with Clear Cell Renal Cell Carcinoma: A Comprehensive Analysis

**DOI:** 10.1155/2023/3898610

**Published:** 2023-04-06

**Authors:** Lin Hou, Xinyue Liu

**Affiliations:** Operating Room, West China Hospital, Sichuan University, West China School of Nursing, Chengdu, China

## Abstract

On a global scale, renal cell carcinoma (RCC) is the second most common form of cancer and the 10th leading cause of cancer-related deaths. There are about 70% of cases of RCC that are clear cell renal cell carcinomas (ccRCCs). This study explores possible targets for immune therapy in patients with RCC. In the recent years, immunotherapy has been applied to RCC patients. In order to identify genes that are closely associated with immune cells, a weighted gene coexpression network analysis (WGCNA) was conducted. A close association was found between genes involved in MEred and M0 macrophages, M1 macrophages, and M2 macrophages. A prognostic prediction model is subsequently developed by incorporating the OS and the expression level of key genes from the RCC cohort into a univariate COX regression analysis, a multivariate COX regression analysis, and a combined COX regression analysis. We finally discovered that 6 genes are closely associated with the prognosis of RCC patients, including SLC16A12, SLC2A9, IGF2BP2, EMX2, ANK3, and METTL7A. The survival analysis proved the prognostic prediction value of the model. The 1-year, 3-year, and 5-year AUC of ROC curves are 0.759, 0.723, and 0.733, respectively. For clinical ROC curves, the AUC score for risk score, stage, grade, and *T* stage is 0.759, 0.824, 0722, and 0.736, respectively. The nomogram was constructed for better prognosis prediction of RCC patients. In addition, GSVA and GO enrichment analysis was performed to explore the potential pathways that are closely associated with genes involved in the prognostic prediction model. Accordingly, our study demonstrates that immune cells play a crucial role in RCC infiltration. The development of a prognostic prediction model is a potential new prognostic biomarker and potential immunotherapy target for tumors.

## 1. Introduction

With an estimated 430,000 new cases and 180,000 deaths each year, renal cell carcinoma (RCC) is one of the top ten causes of cancer-related deaths worldwide [1]. RCC develops from the renal tubular epithelial cells that line the proximal convoluted tubules, the small conduits that transport urine in the kidney [2]. RCC has 15 subtypes, each with its own genetic and epigenetic characteristics, according to the 2012 consensus meeting of the International Society of Urology Pathology (ISUP) [3]. The predominant subtype of RCC is clear cell renal cell carcinoma (ccRCC), making up nearly 70% of all cases of RCC [4]. Approximately 50% of RCCs are discovered incidentally, and a quarter of those patients are diagnosed with metastatic disease [5]. Thirty percent of the patients who undergo nephrectomy for RCC recur and develop metastatic renal cell carcinoma (mRCC), which is considered to be at high risk of death from RCC [6]. In addition to its high morbidity and mortality rates, mRCC has only an 18% five-year survival rate. It is inherently difficult to treat RCC with conventional cancer treatments, such as chemotherapy and radiotherapy [7].

In the past decade, immunotherapy trials targeting various solid tumors have increased dramatically. Cancer immunotherapy has advanced beyond understanding the dialogue between cancer and the immune system to predict cancer prognoses [8]. Surgical removal of solid tumors, followed by chemotherapy and/or radiation therapy, remains the primary treatment for many solid tumors [9]. However, immunotherapy continues to improve patient survival rates rapidly. With the development and emergence of several novel drugs and combinations, medical oncology has made remarkable progress in the past decade. The immune checkpoint monoclonal antibodies are key components of these therapeutic approaches [10]. Multiple malignancies have been treated with immune checkpoint monoclonal antibodies that have achieved remarkable results [11]. Recently, immunotherapy for metastatic melanoma, urothelial carcinoma, nonsmall cell lung cancer, and renal cell carcinoma has revolutionized, reporting unprecedented response rates and survival rates [12]. There is no doubt that immune checkpoint monoclonal antibodies have revolutionized the treatment of many hematological and solid tumors. Current challenges remain in identifying specific molecular and histological biomarkers that predict the immunotherapy response [13]. As a result, the identification of predictive biomarkers for the selection of immune checkpoint monoclonal antibody therapy represents an extremely active area of clinical research [14]. It should be noted, however, that while immunotherapy appears promising for many solid tumors, relatively little progress has been made in RCC [15]. Over the last decade, RCC treatment has undergone significant changes. Aside from surgical resection, there are few effective treatment options for RCC. As a result of current developments in systemic therapy, RCC patients can now receive a number of regimens, including bevacizumab, which inhibits VEGF signaling, as well as mTOR inhibitors and immune checkpoint inhibitors (ICIs) [16]. Patients with metastatic mRCC are currently treated with an ICI-based combination as the standard first-line treatment. While ICI-based combination therapy has greatly improved RCC patient outcomes, most patients still have primary resistance [17]. In order to treat patients with RCC effectively, new therapeutic strategies need to be developed. The established immune reactivity of RCC makes immunotherapy-based drugs a promising treatment option [18].

Recently, in silico analysis has been used in many areas, including cancer prognostic prediction, cancer immunology response, drug sensitivity, and gene mutation analysis. In this work, we aim to explore the relationship between immune cell infiltrations and RCC. In addition, the potential relationship between immunotherapy and RCC patients was also explored. Our research provided a promising direction for the treatment of RCC.

## 2. Methods

### 2.1. Data Downloading

More than 20,000 primary cancer samples and matched normal samples were molecularly characterized by the Cancer Genome Atlas (TCGA, https://www.cancer.gov/about-nci/organization/ccg/research/structural-genomics/tcga), a landmark cancer genomics project. TCGA generated more than 2.5 petabytes of genetic, epigenomic, transcriptomic, and proteomic information in the next several years. Genotype-Tissue Expression is the full name of the GTEx database, which collects sequencing data from the organizations of normal individuals. In this work, the transcriptome data, as well as the clinical characteristics of prostate cancer patients, were downloaded from TCGA and GTEx databases.

### 2.2. Immune Cell Infiltration

A CIBERSORT algorithm was used to analyze RNA-seq data obtained from RCC patients in different subgroups to determine the relative proportions of 22 immune infiltrating cells. To determine the relationship between gene expression and immune cell infiltration, Spearman correlation analysis was performed. Statistically significant results were those with a *P* value of less than 0.05. TIMER (https://cistrome.shinyapps.io/Timer/) is a tool that allows systematic analysis of immune infiltration in cancers. In the current study, TIMER was analyzed to determine the relationship between immune cell infiltration and expression levels.

### 2.3. Weighted Gene Coexpression Network Analysis (WGCNA)

In WGCNA, correlation patterns between genes in different microarray samples are analyzed to study associations between genomes and clinical features. The first step in determining a gene's correlation is to calculate its correlation coefficient. A threshold for screening determines whether two genes have similar expression patterns. Genes above the threshold are considered similar. Using the correlation coefficient between the genes, the second step is to build a hierarchical clustering tree. The clustering tree shows different gene modules as different branches, and the colors show different modules as different colors. Based on their weighted correlation coefficients, genes with similar expression patterns are grouped into modules based on their expression patterns. In order to identify the modular immune cells most associated with these immune infiltrates, WGCNA was performed using the “WGCNA” R package (https://cran.rproject.org/web/packages/wgcna/index.html).

### 2.4. Construction of the Prognostic Prediction Model Based on the RCC Cohort

Members of the module (MM) represent gene expression profiles that are correlated with genes known to be part of the module. Afterwards, we performed univariate analyses for each gene in the module, identifying significantly associated genes with prognosis. With the “glmnet” *R* package, we used the least absolute shrinkage and selection operator (LASSO) Cox regression to further narrow down candidate immunorelated prognostic biomarkers. A bivariate model with nonzero coefficients identified immune-related genes, and samples were divided into low-risk and high-risk groups using the “survminer” *R* package. In addition, survival analysis was also performed in *R*. ROC analysis was conducted using the *R* package “survival ROC.” The AUC (area under the ROC curve) value was used to evaluate the prognostic value of the ROC curve.

### 2.5. Enriched Pathway Analysis Based on the Key Genes

Analyses of GO function annotations based on the GO database (https://geneontology.org/page/go-database) and analyses of KEGG pathway annotations based on the KEGG database (https://www.kegg.jp/kegg/ko.html) were performed to explore the potential pathways that are closely associated with selected genes. The GO enrichment analysis has consisted of the biological process (BP), the molecular function (MF), and the cellular component (CC). As part of this study, the enrichment of gene sets was assessed using GSVA, a method that is nonparametric and unsupervised. By scoring gene sets of interest comprehensively, gene-level changes in this analysis are converted to pathway changes and the biological function of the sample is then determined. A database of molecular signatures was used to retrieve gene sets for this study. The GSVA algorithm was used to assess potential biological functional changes in various samples.

### 2.6. Statistical Analysis

We used the *R* software to conduct all statistical analyses. A *P* value less than 0.05 was considered statistically significant on both sides of the test.

## 3. Results

### 3.1. Exploration of the Module Genes That Are Closely Associated with Macrophages

**Figure 1:**
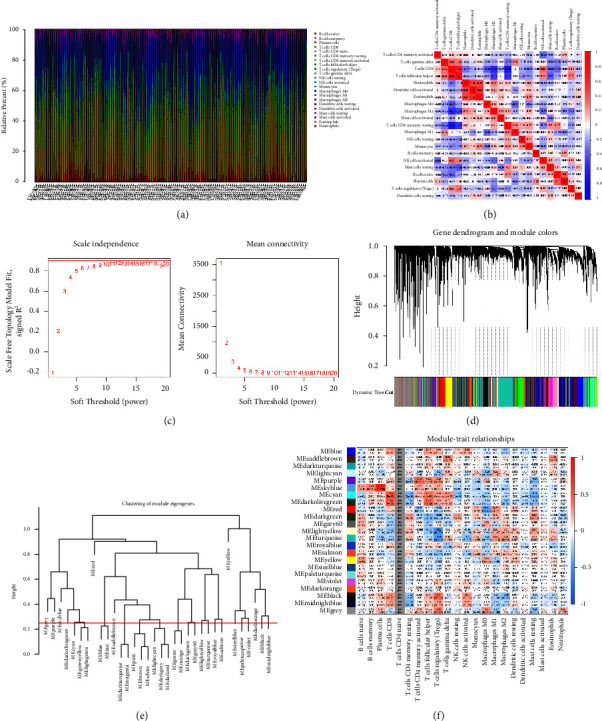
(a) The heatmap demonstrated the immune cell infiltration between RCC tissues and normal renal tissues; (b) the correlation analysis between different immune cells; (c) the threshold for screening determines whether two genes have similar expression patterns; (d, e) the clustering tree shows different gene modules as different branches, and the colors show different modules as different colors; (f) the results of WGCNA shows the correlation between module genes and immune cells.

First, in order to figure out the level of immune cells in all RCC patients from TCGA database, we performed the immune cell infiltration analysis based on the CIBERSORT algorithm. The heatmap reveals the different expression levels of immune-related cells in all RCC cohorts (Figure 1(a)). Subsequently, we evaluate the correlation between different immune-related cells. For the majority of the immune-related cells, a negative relationship has been discovered (Figure 1(b)). Then, in order to obtain molecule genes that are closely associated with certain immune cells, we performed WGCNA. For WGCNA, the soft threshold of 9 is recognized (Figures 1(c)–1(e)). After building a hierarchical clustering tree, we finally obtain a total of 23 gene modules, including MEblue, MEsaddlebrown, MEdarkturquoise, MElightcyan, MEpurple, MEskyblue, MEcyan, MEdarkolivegreen, MEred, MEdarkgreen, MEgrey60, MElightyellow, MEturquoise, MEroyalblue, MEsalmon, MEyellow, MEsteelblue, MEpaleturquoise, MEviolet, MEdarkorange, MEblack, MEmidnightblue, and MEblue (Figure 1(f)). The further analysis discovered that genes involved in MEblue are closely associated with plasma cells. In addition, regulatory *T* cells are closely associated with MEblack genes. For macrophages, we have discovered that both *M*0 macrophages, *M*1 macrophages, and *M*2 macrophages are highly correlated with the genes involved in MEred.

### 3.2. Construction of the Prognostic Prediction Model Based on the Genes That Are Associated with Macrophages

**Figure 2:**
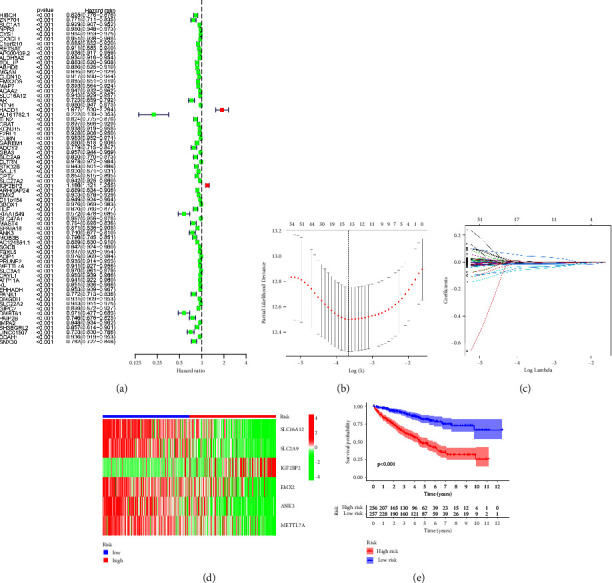
(a) The univariate COX regression analysis based on the overall survival (OS) and the expression of MEred genes; (b, c) the LASSO regression analysis demonstrated that 12 genes are closely associated with the prognosis of RCC patients; (d) the heatmap demonstrated the different expression level of 6 prognosis-related genes between the low- and high-risk groups; (e) the survival analysis between the low- and high-risk groups.

**Figure 3:**
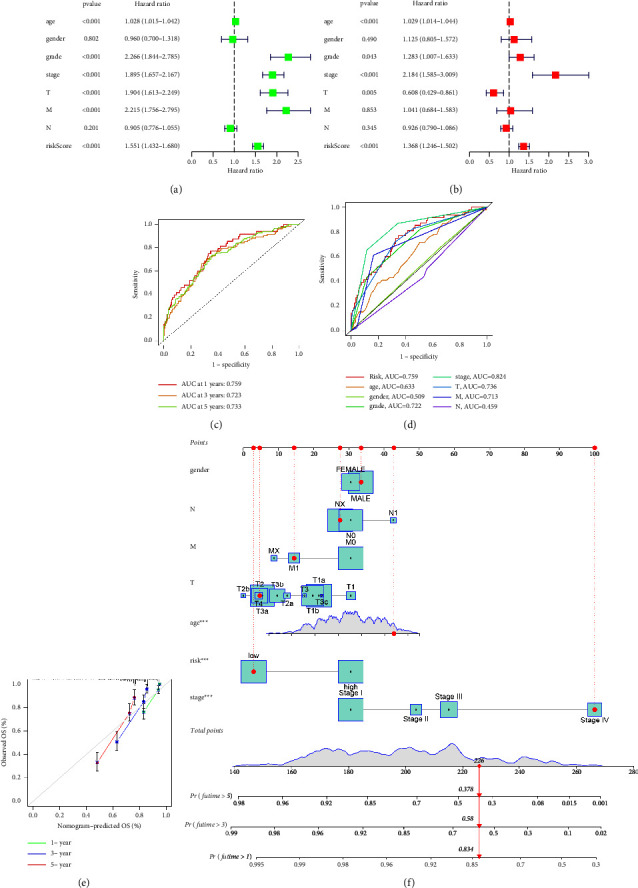
(a) The univariate independent prognostic analysis based on the clinical characteristics and the risk score; (b) the multivariate independent prognostic analysis based on the clinical characteristics and the risk score; (c) the time-dependent ROC curve shows the 1-year, 3-year, and 5-year prognostic prediction value in RCC cohort; (d) the ROC curve demonstrated the prognostic prediction value of the risk score and clinical characteristics; (e) the calibration curve demonstrated that the nomogram shows a good predictive value for the prognosis of RCC patients; (f) the build of nomogram based on the risk score and clinical characteristics.

Based on the previous analysis, we found that genes involved in MEred module are considered to be closely associated with macrophages. Then, we performed the univariate COX regression analysis based on the overall survival (OS) and the expression of MEred genes. The results demonstrated that a total of 70 genes were closely associated with the prognosis of RCC patients (Figure 2(a)). Furthermore, LASSO regression analysis demonstrated that 12 genes (ZNF704, CYS1, AP000439.2, SLC16A12, HACD1, CRAT, F2RL1, SLC2A9, IGF2BP2, EMX2, ANK3, and METTL7A) were considered as prognosis-related genes (Figures 2(b) and 2(c)). Finally, multivariate COX regression analysis demonstrated that 6 genes were involved in the prognostic prediction model, including SLC16A12, SLC2A9, IGF2BP2, EMX2, ANK3, and METTL7A (Figure 2(d)). The survival analysis revealed that RCC patients involved in the high-risk group are associated with poorer OS (Figure 2(e)). Subsequently, we conduct univariate and multivariate independent prognostic analysis based on the clinical characteristics and the risk score. The univariate independent prognostic analysis showed that grade, stage, *T* stage, *M* stage, and risk score are the independent prognostic factors of RCC patients (Figure 3(a)). For multivariate independent prognostic analysis, grade, stage, and risk score are the independent prognostic factors of RCC patients (Figure 3(b)). For both univariate and multivariate independent prognostic analysis, the risk score based on the expression level of SLC16A12, SLC2A9, IGF2BP2, EMX2, ANK3, and METTL7A are considered to be highly correlated with the prognosis of RCC patients. Furthermore, we performed the time-dependent ROC curve. The 1-year, 3-year, and 5-year AUC of ROC curves is 0.759, 0.723, and 0.733, respectively (Figure 3(c)). For clinical ROC curves, the AUC score for risk score, stage, grade, and *T* stage is 0.759, 0.824, 0722, and 0.736, respectively (Figure 3(d)). The results revealed that the model showed a good predictive value for RCC patients. Subsequently, in order to construct a better predictive tool for RCC patients, we build a nomogram based on clinical characteristics and risk scores (Figure 3(f)). The calibration curve demonstrated that the nomogram shows the good predictive value for the prognosis of RCC patients (Figure 3(e)).

### 3.3. Exploration of the Correlation between Clinical Characteristics and the Risk Score

**Figure 4:**
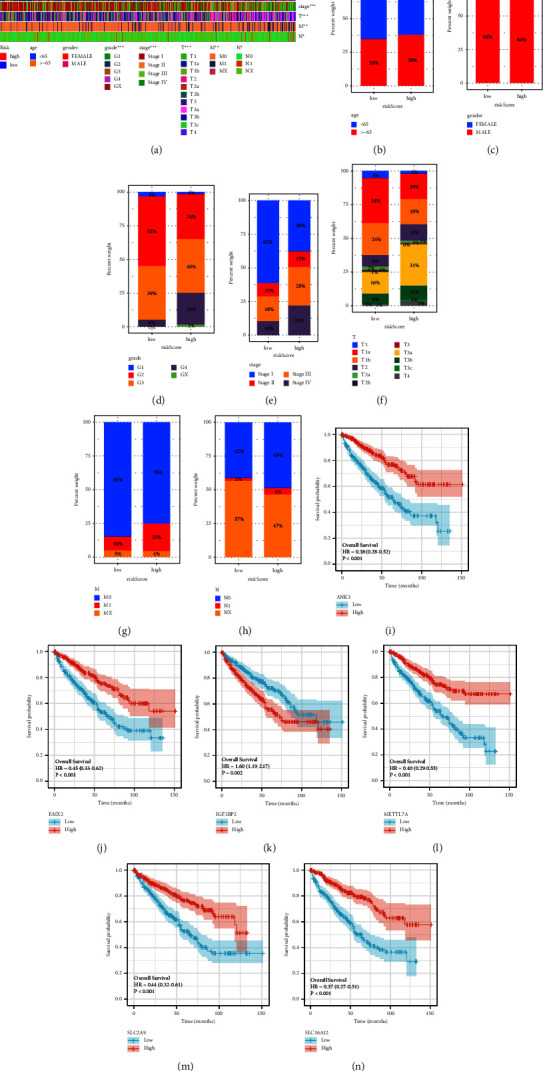
(a) The heatmap shows the correlation between the risk score and clinical characteristics. The histogram reveals the relationship between risk score and age (b), gender (c), grade (d), stage (e), *T* stage (f), *M* stage (g), and *N* stage (h) The survival analysis between the low- and high-expression groups of ANK3 (i), EMX2 (j), IGF2BP2 (k), METTL7A (l), SLC2A9 (m), SLC16A12 (n).

On the basis of the risk score involved in the prognostic prediction model, the RCC patients were divided into low- and high-risk groups. The heatmap was performed to show the correlation between the risk score and clinical characteristics, including age, gender, grade, stage, *T* stage, *N* stage, and *M* stage (Figure 4(a)). The results revealed that grade, stage, *T* stage, *N* stage, and *M* stage are closely associated with the risk score with a *P* value less than 0.05, while age and gender show no obvious relationship with a risk score. For RCC patients involved in a high-risk group, the higher risk score is related to the high grade, stage, *T* stage, and *M* stage. However, for the *N* stage, the higher risk score is associated with the lower *N* stage (Figures 4(b)–4(h)). In addition, we also evaluate the prognostic value of six prognosis-related alone in RCC patients. The results demonstrated that the high-expression level of ANK3, EMX2, METTL7A, SLC2A9, and SLC16A12 is associated with better OS, while the high expression of IGF2BP2 is associated with poorer OS (Figures 4(i)–4(n)).

### 3.4. The Risk Score Is Also Strongly Correlated with Immune Cell Infiltration in the RCC Cohort

**Figure 5:**
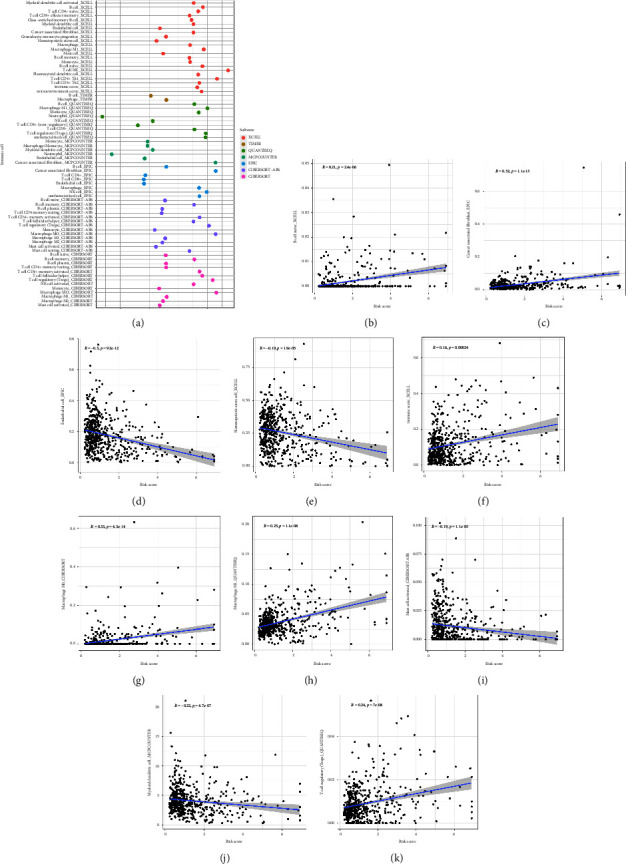
(a) The correlation between the risk score and the immune score, including naïve *B* cell (b); cancer-associated fibroblast (c); endothelial cell (d); hematopoietic cell (e); immune score (f); *M*0 macrophage (g); *M*1 macrophage (h); activated mast cell (i); myeloid dendritic cell (j); regulatory *T* cell (k).

Subsequently, we evaluated the potential relationship between the risk score and immune cell infiltration in the RCC cohort. The results reveal that the risk score is positively correlated with naïve *B* cell, cancer-associated fibroblast, immune score, *M*0 macrophages, *M*1 macrophages, and regulatory *T* cell. However, the endothelial cell, hematopoietic stem cell, activated mast cell, and myeloid dendritic cell are negatively correlated with a risk score (Figures 5(a)–5(k)).

### 3.5. Exploration of the Potential Pathways That Are Closely Associated with the Key Genes

**Figure 6:**
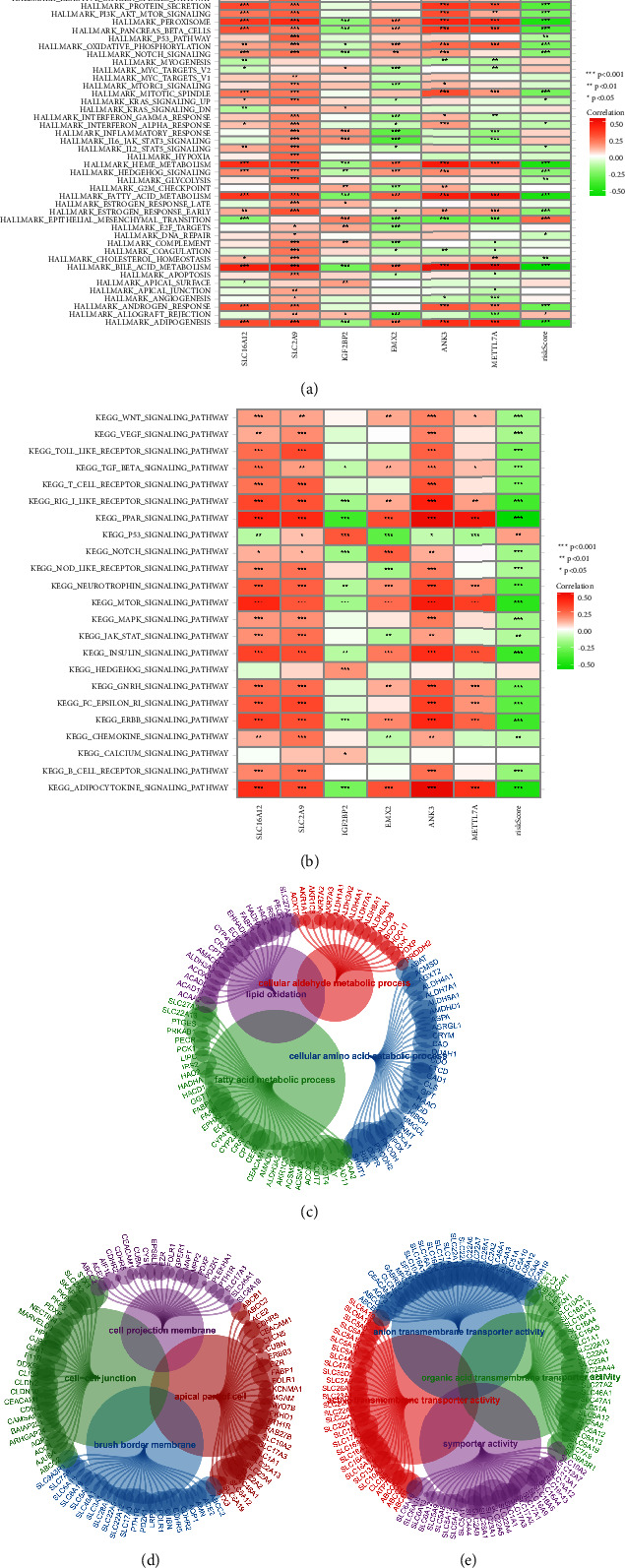
(a) The results of GSVA based on the HALLMARK enrichment analysis; (b) the results of GSVA based on HALLMARK enrichment analysis; (c) the GO BP enrichment analysis based on genes involved in the MEred module; (d) the GO CC enrichment analysis based on genes involved in the MEred module; (e) the GO MF enrichment analysis based on genes involved in the MEred module.

We first evaluate the potential pathways of genes involved in the risk score, such as SLC16A12, SLC2A9, IGF2BP2, EMX2, ANK3, and METTL7A, by GSVA enrichment analysis. For HALLMARK enrichment pathways, the results revealed that SLC2A9 is positively correlated with the majority of the pathways, including bile acid metabolism, fatty acid metabolism, heme metabolism, adipogenesis, and androgen response. However, the risk score is negatively correlated with the majority of the pathways, including xenobiotic metabolism, protein secretion, peroxisome, heme metabolism, and fatty acid metabolism (Figure 6(a)). For KEGG enrichment pathways, the results revealed that SLC16A12 and SLC2A9 are positively correlated with PPAR signaling, mTOR signaling pathway, ERBB signaling pathway, VEGF signaling pathway, WNT signaling pathway, and *B* cell receptor signaling pathway. The risk score is also negatively associated with the WNT signaling pathway, GnRH signaling pathway, MTOR signaling pathway, insulin signaling pathway, and JAK-STAT signaling pathways (Figure 6(b)). For all genes involved in the MEred module, we then performed GO enrichment analysis. The GO BP is closely associated with lipid oxidation, fatty acid metabolic process, cellular amino acid catabolic process, and cellular aldehyde metabolic process (Figure 6(c)). For GO CC, cell-cell junction, cell projection membrane, apical part of the cell, and brush border membrane are the most enriched pathways (Figure 6(d)). In terms of BP MF, the anion transmembrane transporter activity, active transmembrane transporter activity, organic acid transmembrane transporter activity, and symporter activity are closely associated with genes involved in MEred (Figure 6(e)).

## 4. Discussion

There is an increase in the incidence of RCC over time, with the incidence of the disease being sixth for men and tenth for women, accounting for 5% and 3% of all malignancies, respectively [19]. In patients with locally or locally advanced disease, surgery remains the cornerstone of treatment, yet a significant number of patients experience disease recurrences [20]. As a result of the poor prognosis of these individuals, studies have been conducted to evaluate what can be achieved with adjuvant and neoadjuvant medical therapy in addition to surgery alone [21]. In the recent years, with the development of bioinformatics analysis methods, in silico analysis has been applied in many fields of medical research, including disease diagnosis, immunotherapy, and therapy targets [22]. In this work, we aim to explore the potential relationship between RCC and immune response. First, by performing WGCNA, we discovered that many module genes are associated with different immune-related cells. In addition, we also discovered that genes involved in MEred are closely associated with both *M*0 macrophages, *M*1 macrophages, and *M*2 macrophages. Therefore, the genes involved in MEred are selected for further analysis. In the recent years, macrophages have been found to play a key role in the development of RCC. As is reported by a former research study, *M*1 macrophage fraction correlates significantly with the stage and the tumor histological grade of RCC. Additionally, a high expression of FCER1G in ccRCC is closely linked to infiltration of the tumor microenvironment, which inhibits *T* cell proliferation and activation. Combined FCER1G expression levels and macrophage biomarker CD68 expression levels may be promising postoperative prognostic indicators in patients with ccRCC [23]. In terms of another study, by simulating a large number of infiltrating macrophages, HK3 activates the ccRCC microenvironment and promotes the microenvironmental signature of active antitumor immunity [24].

Subsequently, based on the OS and the expression level of key genes in the RCC cohort, we then construct the prognostic prediction model. The results demonstrated that 6 genes are closely associated with the prognosis of RCC patients, including SLC16A12, SLC2A9, IGF2BP2, EMX2, ANK3, and METTL7A. Both the ROC curve and survival analysis proved that the model shows the good predictive value for RCC patients. In addition, the univariate and multivariate independent prognostic analysis demonstrated that the prognostic prediction model, as well as the clinical characteristics, is the independent risk factor for RCC patients. In order to further construct a better model for the prediction of RCC prognosis, we then build a nomogram based on the risk score and clinical characteristics. We further discovered that SLC16A12 and IGF2BP2 have been studied by researchers in the RCC cohort. In a previous study, SLC16A12 was found to be a poor prognostic factor in ccRCC patients. These findings suggest that SLC16A12 might be a potential biomarker and a treatment target for ccRCC [25]. For IGF2BP2, ccRCC progression and metastasis can be inhibited by Circ-TNPO3 by binding directly to the IGF2BP2 protein and destabilizing the SERPINH1 mRNA [26]. In addition, our study also found that the high-expression level of SLC16A12 is associated with better OS of RCC patients, while the high expression of IGF2BP2 is associated with poorer OS of RCC patients.

Furthermore, we found that the risk score is also closely associated with many immune cells, such as naïve *B* cell, cancer-associated fibroblasts, *M*0 macrophages, *M*1 macrophages, and regulatory *T* cells, which may provide the targets for the immunotherapy for RCC patients. The clinical outcome of RCC is closely related to immune responses [27]. As tumor-infiltrating immune cells regulate cancer progression and show a potential prognostic value, they form an ecosystem within the tumor microenvironment [28]. A number of studies have demonstrated that regulatory T cells can effectively inhibit the proliferation of effector *T* cells in RCC [29]. In addition to macrophages, tumor-associated macrophages play an important role in the development of tumors. Recently, treatments targeting PD-1, CTLA-4, and other immune checkpoint pathways have significantly improved the outcomes for patients with mRCC [30].

Finally, we explore the potential pathways that are closely associated with the key genes. The results of GSVA reveal that the risk score is highly correlated with the WNT signaling pathway, GnRH signaling pathway, MTOR signaling pathway, insulin signaling pathway, and JAK-STAT signaling pathways. On the basis of the former research, these pathways play an important role in the development of RCC [31]. A basic study found that the mTOR signaling pathway is activated by ITPKA1 and promotes renal cell carcinoma growth, migration, and invasion [32]. Also, by inhibiting the PI3K/Akt/mTOR signaling pathway, bufalin inhibits renal cell carcinoma proliferation and metastasis [33].

However, there are some limitations in the bioinformatics analysis. First of all, the limited data from the online dataset may lead to different results of the analysis [34]. In addition, the single cohort may also lead to bias [35–38]. Therefore, more analysis should be involved to further promote the quality of the analysis.

## 5. Conclusion

In this work, by exploring the key genes that are closely associated with immune cells, we discovered the genes that are closely associated with macrophages. In addition, a 6-gene-based prognostic prediction model shows a good predictive value for the patients involved in the RCC cohort. Finally, the potential pathways of the key genes may provide new insights for RCC immunotherapy.

## Data Availability

The data used to support the findings of this study are included within the article.
